# Relationship between sugarcane culm and leaf biomass composition and saccharification efficiency

**DOI:** 10.1186/s13068-019-1588-3

**Published:** 2019-10-17

**Authors:** K. Hodgson-Kratky, G. Papa, A. Rodriguez, V. Stavila, B. Simmons, F. Botha, A. Furtado, R. Henry

**Affiliations:** 1 Queensland Alliance for Agriculture and Food Innovation, University of Queensland, Brisbane, QLD 4072 Australia; 2 Sugar Research Australia, Brisbane, QLD 4068 Australia; 3 Joint BioEnergy Institute, Emeryville, CA 94608 USA; 4 Sandia National Laboratories, Livermore, CA 94550 USA; 5 Advanced Biofuels and Bioproducts Process Development Unit, Emeryville, CA 94608 USA

**Keywords:** Sugarcane, Lignocellulosic biomass, Biofuel, Pretreatment, Enzymatic hydrolysis, Saccharification efficiency, Xylan, Lignin, S/G ratio

## Abstract

**Background:**

Lignocellulosic biomass is recognized as a promising renewable feedstock for the production of biofuels. However, current methods for converting biomass into fermentable sugars are considered too expensive and inefficient due to the recalcitrance of the secondary cell wall. Biomass composition can be modified to create varieties that are efficiently broken down to release cell wall sugars. This study focused on identifying the key biomass components influencing plant cell wall recalcitrance that can be targeted for selection in sugarcane, an important and abundant source of biomass.

**Results:**

Biomass composition and the amount of glucan converted into glucose after saccharification were measured in leaf and culm tissues from seven sugarcane genotypes varying in fiber composition after no pretreatment and dilute acid, hydrothermal and ionic liquid pretreatments. In extractives-free sugarcane leaf and culm tissue, glucan, xylan, acid-insoluble lignin (AIL) and acid-soluble lignin (ASL) ranged from 20 to 32%, 15% to 21%, 14% to 20% and 2% to 4%, respectively. The ratio of syringyl (S) to guaiacyl (G) content in the lignin ranged from 1.5 to 2.2 in the culm and from 0.65 to 1.1 in the leaf. Hydrothermal and dilute acid pretreatments predominantly reduced xylan content, while the ionic liquid (IL) pretreatment targeted AIL reduction. The amount of glucan converted into glucose after 26 h of pre-saccharification was highest after IL pretreatment (42% in culm and 63.5% in leaf) compared to the other pretreatments. Additionally, glucan conversion in leaf tissues was approximately 1.5-fold of that in culm tissues. Percent glucan conversion varied between genotypes but there was no genotype that was superior to all others across the pretreatment groups. Path analysis revealed that S/G ratio, AIL and xylan had the strongest negative associations with percent glucan conversion, while ASL and glucan content had strong positive influences.

**Conclusion:**

To improve saccharification efficiency of lignocellulosic biomass, breeders should focus on reducing S/G ratio, xylan and AIL content and increasing ASL and glucan content. This will be key for the development of sugarcane varieties for bioenergy uses.

## Background

Concerns regarding the depletion of fossil fuel reserves and the environmental consequences of their use have motivated the development of renewable fuels and feedstocks with low net carbon emissions [1]. Lignocellulosic biomass derived from agricultural residues is recognized as a promising raw material for fuel production because it is sustainable and highly abundant. In tropical and sub-tropical regions, waste from sugar production is the one of the most significant sources of biomass. Worldwide, there are about 1.8 billion Mg of sugarcane processed annually [2] and over 500 kg of bagasse and leaf trash are produced from each megagram [3]. This equates to more than 900 million Mg of biomass that could be used to replace fossil oil, each year. Additionally, dedicated bioenergy crops, which are grown solely for their biomass, are under development to increase the quantity of lignocellulosic material available for biofuel production [4]. Ideally, these crops will be fast-growing and adapted to marginal land, where food crops cannot grow, so that they do not compete with food production. Sugarcane is considered among the most promising candidates for biomass production because it is one of the highest-yielding crops due to its efficient use of solar energy [5].

Lignocellulosic biomass is primarily composed of cellulose, hemicellulose and lignin, which are bound together to form the secondary cell wall. Cellulose is made up of linear chains of repeating glucose units arranged into semi-crystalline microfibrils, while hemicelluloses are branched heteropolymers consisting of pentose and hexose sugars. Xylans are the most abundant class of hemicelluloses present in sugarcane [6]. Their structure comprises a linear backbone of xylose residues to which side chains of arabinose, glucuronic acid and acetic acid are often attached [7]. The xylan polymers form hydrogen bonds with the cellulose microfibril imparting strength to the cell wall [8]. Lignin is a heterogeneous phenolic polymer mainly composed of *p*-hydroxyphenyl (H), guaiacyl (G), and syringyl (S) phenylpropanoid units that are derived from the monolignols *p*-coumaryl, coniferyl, and sinapyl alcohol, respectively [9]. Lignin is linked to xylan by electrostatic interactions (non-covalent bonds) [10] and is crucial for strengthening and waterproofing the cell wall to maintain structural stability and facilitate water transport [9].

The production of fuels and chemical feedstocks from lignocellulosic biomass requires that the cellulose and hemicelluloses are converted into monomeric sugars by saccharification and then microbially fermented. Saccharification is achieved using cellulase and hemicellulase enzymes which hydrolyze the glycosidic linkages between the monosaccharides. However, without prior pretreatment, these linkages are relatively inaccessible to the hydrolases because of the strong interactions between the cell wall constituents that create a recalcitrant matrix. To improve the efficiency of saccharification, the structure and composition of the biomass are often modified using harsh physical, chemical or thermochemical pretreatments [11]. Dilute acid (DA) and hydrothermal (HT) pretreatments are two examples of well-established pretreatments commonly used for sugarcane biomass [12–15]. They both create an acidic environment which loosens the cell wall by targeting the removal of xylan and are favored over other pretreatments because they are inexpensive and efficient in hydrolyzing lignocellulose [16]. Pretreatment using ionic liquids (IL) is emerging as a promising method because a large proportion of the lignin present in the biomass is solubilized allowing for high sugar yields from saccharification [17]. Another advantage is that they are well suited for relatively high concentrations of biomass during pretreatment which are necessary for an economically viable industrial operation [18].

Although a high saccharification efficiency can be achieved after pretreatment, this step represents a significant amount of the total operational expense and economic feasibility is one of the main challenges facing lignocellulosic biofuel production. It was recently estimated that lignocellulosic biofuel is more than twice as expensive as petroleum fuel to produce [19] and that pretreatment and saccharification enzymes account for 30–50% of the operational costs [20–22]. One approach to improve the economics of production is to breed crops that have less recalcitrant cell walls. Biomass that is more amenable to saccharification will reduce the amount of energy, chemicals, and enzymes required, and therefore bring the costs down.

Many research studies have sought to understand cell wall recalcitrance and have identified that it is a complex trait controlled by a number of factors. Lignin has been by far the most prominently featured in studies as the primary cause of recalcitrance [23–27]. This is because its linkages with hemicellulose create a condensed lignocellulosic matrix which obstructs cellulose and hemicellulose breakdown. Lignin can also irreversibly bind cellulase enzymes preventing them from cleaving the glycosidic bonds of cellulose [28, 29]. Research has shown that biomass low in lignin has improved enzymatic hydrolysis yields [26, 30]. For instance, a 6% reduction in lignin content increased saccharification efficiency in sugarcane up to 23% compared to control plants [31].

While lignin is the most universally recognized cause of recalcitrance, the importance of other biomass components has been increasingly reported [32–36]. For instance, several studies have shown that removing xylan increases the porosity of the biomass and results in higher conversion of cellulose during saccharification [37–41]. Hydroxycinnamic acids, ferulic acid (FA) and *p*-coumaric acid (CA) [23, 42, 43] and lignin composition (S/G ratio) [31, 44, 45] are other factors that have been implicated in influencing recalcitrance because they affect the strength and abundance of the cross-links between lignin and xylan [10, 46]. Moreover, a high abundance of mixed linkage β-d-glucan (MLG), a hemicellulose that is loosely bound to the cell wall, has been associated with improved hydrolysis efficiency [33]. This is because it is composed of glucose units and is easily accessible and hydrolysable by enzymes, thus contributing to the total glucose released from saccharification. Furthermore, the degree of crystallinity in the cellulose is also known to affect saccharification efficiency because the fibrils in crystalline regions are tightly bound to each other which further obstructs enzymes from accessing them [47].

Despite the vast amount of research focused on elucidating the relationship between biomass composition and saccharification efficiency, there is still much to understand. For instance, most studies are based on examination of biomass material derived from a single pretreatment [23–25]. Yet, many biomass pretreatments are available and each modify the structure and composition in a unique way [48]; so, studies that were limited to one method may not have identified relationships between traits that were representative for other pretreatments. Therefore, understanding the relationships between biomass composition and cellulose conversion efficiency after various pretreatments was the main objective of this study. Cell wall components were quantified in leaf and culm tissues of seven sugarcane genotypes and their impact on saccharification efficiency after HT, DA and IL pretreatments was assessed.

## Results and discussion

### Compositional analysis of untreated leaf and culm tissues

The biomass composition of the seven genotypes is presented on a total dry matter basis of unextracted material in Tables 1 and 2. The fiber content in the leaf ranged from 46.3 to 56.3% (Table 1) and was higher and less variable than that of the culm tissues, which ranged from 21.9 to 40.5% (Table 2). This is not surprising because fiber and sugar contents are inversely related [49] and total sugar content in the culm was approximately 10 times higher than that in the leaf tissue. High-fiber genotypes had low culm sugar content (< 38%) whereas low-fiber genotypes were high in sugar (> 47%) (Table 2). Moreover, sucrose was the most abundant sugar comprising 23.6–51.7% and 1.6–4.3% of total dry matter in the culm and leaf, respectively.

**Table 1:** Composition of unextracted, raw sugarcane leaf tissues (% dry matter, DM)

Genotype	FIJI_62	MQ239	Q124	Q208	QBN13-10020	SRA1	SRA5
Total fiber^a^	51.5 ± 2.1	55.9 ± 6.3	56.3 ± 5.2	54.2 ± 3.7	48.7 ± 1.2	46.3 ± 2.9	53.4 ± 3.6
Glucan	16.7 ± 0.9	22.3 ± 4.2	23.7 ± 3.5	22.5 ± 1.7	18.3 ± 0.9	17.9 ± 1.7	19.3 ± 2.6
Xylan	12.7 ± 0.7	17.2 ± 2.6	17.1 ± 2.1	15.5 ± 1.1	13.9 ± 0.3	12.9 ± 0.4	16.1 ± 1.8
AIL	19.1 ± 2.2	13.1 ± 1	12.2 ± 0.8	13 ± 1	13.5 ± 0.4	12.7 ± 0.5	15.1 ± 1.2
ASL	2.9 ± 0.3	3.3 ± 0.5	3.4 ± 0.4	3.2 ± 0.1	2.8 ± 0.1	2.8 ± 0.6	2.9 ± 0.2
Total sugar^a^	6.3 ± 1.7	4 ± 0.8	3.6 ± 0.5	3.9 ± 0.5	3.4 ± 0.6	4.2 ± 0.1	4 ± 1
Sucrose	4.3 ± 1.5	2.4 ± 0.7	1.6 ± 0.3	2.4 ± 0.6	2 ± 0.6	2.8 ± 0.1	2 ± 0.2
Glucose	0.97 ± 0.18	0.78 ± 0.13	1.0 ± 0.07	0.80 ± 0.08	0.66 ± 0.03	0.71 ± 0.15	0.83 ± 0.30
Fructose	1.0 ± 0.07	0.77 ± 0.07	0.97 ± 0.17	0.73 ± 0.02	0.71 ± 0.03	0.73 ± 0.06	1.2 ± 0.83
CA	1.6 ± 0.1	1.4 ± 0	1.4 ± 0	1.2 ± 0.1	1.4 ± 0.1	1.5 ± 0.1	1.6 ± 0.1
FA	0.95 ± 0.07	1.17 ± 0.05	1.06 ± 0.05	0.89 ± 0.1	0.99 ± 0.03	0.94 ± 0.02	0.76 ± 0.11
Ash	3.7 ± 0.4	3.1 ± 0.2	6.1 ± 0.9	3.8 ± 0.3	5.6 ± 0.6	3.8 ± 0.2	2.9 ± 0.2
MLG	0.53 ± 0.03	0.6 ± 0.08	0.68 ± 0.07	0.69 ± 0.07	0.32 ± 0.04	0.66 ± 0.09	0.47 ± 0.02
Cellulose CrI^b^	40	–	–	–	42	44	–

Means ± standard deviation; percentage of fiber components (glucan, xylan, AIL and ASL) based on their original amount in unextracted biomass calculated from alcohol-insoluble residue (AIR) amounts (i.e. (% component in AIR/100) × (100 − % extractives)

*AIL* acid-insoluble lignin, *ASL* acid-soluble lignin, *CA* coumaric acid, *FA* ferulic acid, *MLG* mixed linkage β-d-glucan

^a^Total fiber = glucan + xylan + AIL + ASL; total sugar = sucrose + glucose + fructose

^b^Crystallinity index (CrI) of cellulose determined by X-ray diffraction performed on subset of 10 samples

**Table 2:** Composition of unextracted, raw sugarcane culm tissues (% dry matter, DM)

Genotype	FIJI_62	MQ239	Q124	Q208	QBN13-10020	SRA1	SRA5
Total fiber^a^	25.3 ± 2.0	30 ± 4.5	21.9 ± 1.9	27.3 ± 5.9	40.5 ± 3.1	25.7 ± 4.1	35.1 ± 5.7
Glucan	10.2 ± 0.6	11.1 ± 2.8	8.6 ± 1	10.8 ± 2.8	16 ± 1.7	10.4 ± 1.9	14.8 ± 3.5
Xylan	7.4 ± 0.7	8.0 ± 1.4	6.0 ± 0.7	7.4 ± 1.9	11.1 ± 1.1	7.3 ± 1.2	9.7 ± 1.9
AIL	6.7 ± 0.5	9.6 ± 0.3	6.2 ± 0.2	8.0 ± 1	11.4 ± 0.1	6.7 ± 0.9	9.0 ± 0.4
ASL	1.0 ± 0.2	1.2 ± 0.3	1.1 ± 0.1	1.2 ± 0.3	2.0 ± 0.3	1.2 ± 0.2	1.6 ± 0.4
Total sugar^a^	47.1 ± 1.3	37.9 ± 3.1	49.9 ± 4.4	55.5 ± 2	28.6 ± 2.8	52.1 ± 4.5	34.3 ± 0.8
Sucrose	38.4 ± 2.5	30.9 ± 4.4	41.4 ± 2.7	51.7 ± 2.1	23.6 ± 2.8	45.2 ± 5.8	29.6 ± 0.9
Glucose	5.2 ± 0.8	3.9 ± 0.8	5.7 ± 1.7	2.5 ± 0.8	3 ± 0.3	4 ± 1.1	2.8 ± 0.7
Fructose	3.6 ± 0.5	3.0 ± 0.6	2.8 ± 0.5	1.8 ± 0.2	2.0 ± 0.1	2.8 ± 0.4	1.9 ± 0.4
CA	2.5 ± 0.2	2.3 ± 0.1	2.9 ± 0.2	2.6 ± 0.2	2.7 ± 0.2	3.0 ± 0.6	2.8 ± 0.4
FA	0.61 ± 0.05	0.62 ± 0.04	0.78 ± 0.05	0.64 ± 0.03	0.76 ± 0.02	0.73 ± 0.12	0.61 ± 0.03
Ash	1.3 ± 0.1	0.74 ± 0.2	1.1 ± 0.1	1.0 ± 0.3	1.8 ± 0.2	1.2 ± 0.1	1.1 ± 0.2
MLG	0.49 ± 0.06	0.36 ± 0.07	0.33 ± 0.05	0.40 ± 0.04	0.27 ± 0.05	0.71 ± 0.11	0.47 ± 0.04
Cellulose CrI^b^	39	45	46	42	44	41	47

Means ± standard deviation; percentage of fiber components (glucan, xylan, AIL and ASL) based on their original amount in unextracted biomass calculated from alcohol-insoluble residue (AIR) amounts (i.e. (% component in AIR/100) × (100 − % extractives)

AIL: acid-insoluble lignin, ASL: acid-soluble lignin, CA: *p*-coumaric acid, FA: ferulic acid, MLG: mixed linkage β-d-glucan

^a^Total fiber = glucan + xylan + AIL + ASL; total sugar = sucrose + glucose + fructose

^b^Crystallinity index (CrI) of cellulose determined by X-ray diffraction performed on subset of 10 samples

Genotypes with high fiber content (≥ 30%) in the culm were QBN13-10020, SRA5 and MQ239 and low-fiber (< 30%) were Q124, Fiji_62, SRA1 and Q208 (Table 2). The fiber components in the culm and leaf tissue, ranged from 8.6 to 16% and 16.7% to 23.7% glucan (cellulose), 6% to 11.1% and 12.7% to 17.2% xylan (hemicellulose), 6.2% to 11.4% and 12.2% to 19.1% acid-insoluble lignin (AIL) and 1% to 2% and 2.8% to 3.4% acid-soluble lignin (ASL), respectively (Tables 1 and 2). QBN13-10020 culm tissues had the highest amount of each fiber constituent listed. This was expected since QBN13-10020 was selected based on its genetic background to represent the weedy and fibrous phenotype characteristic of the commercial hybrid progenitor, *S. spontaneum*. FIJI_62 was chosen to represent the opposite phenotype (e.g. high sugar, low fiber) found in the other progenitor species, *S. officinarum*. However, it did not have the lowest fiber content compared with the other genotypes likely because they have been intensively bred to increase sucrose and decrease fiber.

CA content was higher in the culm than in the leaf ranging from 2.3 to 3% for the former and 1.2% to 1.6% for the latter (Tables 1 and 2). FA and ash contents, in contrast, were higher in the leaf, accounting for 0.8–1.2% and 2.9–6.9%, compared to the culm which had 0.6–0.8% and 0.7–1.8%, respectively. MLG ranged from 0.27 to 0.71% in culm and leaf tissues. The SRA1 culm tissue contained the most MLG while QBN13-1002 had the least in both tissues. Since MLG is easily extracted, much of it was likely removed during the preparation of the alcohol-insoluble residue (AIR), which was used for simultaneous saccharification and fermentation (SSF), so MLG content was not considered when identifying traits affecting glucan conversion in the later analyses. CrI varied from 39 to 47% in the culm and from 40 to 44% in the leaf which is similar to the values obtained by Costa et al. [33] and Moutta et al. [50]. In contrast, Caliari et al. [51] and Silva et al. [52] found a much greater range in crystallinity (from 50 to 81%) when surveying more than 90 Brazilian genotypes. Since the variability in crystallinity was fairly low in the present study and the difference among the subset of samples analyzed (*n* = 10) was not statistically significant, CrI was not considered in the later analyses. S/G ratios were consistent with the literature [53] and ranged from 1.5 to 2.2 for culm tissues and 0.65 to 1.1 for leaf tissues indicating that S units are predominant in the lignin from the culm; whereas G units are predominant in the lignin from the leaf (Table 3). Interestingly, all genotypes had ratios of approximately 0.7 or 0.8 for the leaf tissue except FIJI_62, which had a ratio of 1.1. This may be a result of its genetic background since it is the only genotype that is not a hybrid. Additionally, other than 4-vinylphenol which is mainly derived from *p*-coumarate and, therefore, not included in the lignin composition determination [53], H units were not detected in either leaf or culm tissue.

**Table 3:** Relative abundances of lignin-derived compounds released after py–GC/MS from unextracted, raw sugarcane culm and leaf tissue

Compound^a^	Origin	FIJI_62	MQ239	Q124	Q208	QBN13-10020	SRA1	SRA5	FIJI_62	MQ239	Q124	Q208	QBN13-10020	SRA1	SRA5
		Culm							Leaf						
2-Methoxy-5-(1-propenyl)phenol	G	0.4	0.3	0.3	0.3	0.5	0.2	0.3	0.4	0.4	0.4	0.3	0.4	0.2	0.3
4-Ethylguaiacol	G	0.5	0.6	0.5	0.6	0.7	0.4	0.5	0.5	0.7	0.8	0.6	0.8	0.5	0.5
4-Methoxy-3-methoxymethyl phenol	G	1.5	1.3	1.6	1.3	2.0	1.2	1.6	0.8	0.8	0.8	0.5	0.9	0.4	1.4
4-Propenylguaiacol	G	1.8	1.8	1.2	1.3	1.9	1.1	1.7	1.6	1.6	1.8	1.3	1.6	0.9	1.3
4-Vinylguaiacol	G	6.4	6.5	6.1	5.0	8.4	3.8	6.3	8.3	9.7	10.1	7.9	9.3	4.0	5.3
Guaiacol	G	2.0	2.5	1.6	1.4	2.4	1.1	1.7	2.1	2.4	2.6	2.0	2.4	2.6	1.5
Isovanillin	G	0.8	0.8	0.9	0.6	1.2	0.4	0.8	0.7	0.7	0.9	0.7	1.0	0.3	0.5
4-Vinylphenol	H	9.1	6.4	12.7	9.1	13.0	5.5	10.2	6.7	5.3	6.5	4.7	6.0	2.7	4.2
4-Allylsyringol	S	5.4	5.4	7.0	6.2	8.5	4.5	6.7	3.9	2.6	2.7	2.0	3.1	2.0	2.1
Acetosyringone	S	0.7	0.8	0.2	0.8	1.2	0.6	0.9	0.7	0.7	0.2	0.6	0.8	0.1	0.6
Syringol	S	4.8	4.6	6.0	5.2	7.5	2.7	5.9	2.4	1.7	2.6	1.9	2.1	1.2	1.4
S/G ratio ± SD		1.6 ± 0.1	1.5 ± 0.1	2.1 ± 0.2	2.2 ± 0	2.0 ± 0.1	1.7 ± 0.27	2 ± 0.2	1.1 ± 0.03	0.77 ± 0.12	0.76 ± 0.18	0.81 ± 0.15	0.85 ± 0.05	0.65 ± 0.15	0.75 ± 0.13

^a^4-vinylguaiacol and 4-vinylphenol were not used to estimate the ratio of syringyl (S) to guaiacyl (G) units (S/G ratio) and *p*-hydroxyphenyl (H) content, respectively, because they predominantly arise from ferulates and *p*-coumarate esters [53]

The genotypes were also compared on a starch and extractives-free basis (AIR samples) (Table 4), and the fiber constituents comprising culm tissues were generally higher and had a smaller range than those calculated on an unextracted basis (Table 2). For instance, AIL and xylan amounts were between 18.3 and 19.8% and 16.3% to 20.8%, respectively (Table 4). Additionally, genotype rankings for each trait in the culm AIR samples were not consistent with the rankings observed when calculated on an unextracted basis. The highest and lowest values for glucan were in SRA5 (31.5%) and MQ239 (22.5%) based on AIR (Table 4) and in QBN12-10020 (16%) and Q124 (8.6%) when calculated on an unextracted basis, respectively (Table 2). In a commercial setting, bagasse, which is free of sugars and other extractives, will likely be used as the feedstock for second-generation ethanol production rather than whole cane. Therefore, the inconsistency and trends of the analytical results of fiber for unextracted and AIR-based material suggest that genotypes should be compared on an extractives-free basis rather than based on the unextracted native feedstock, when developing bioenergy varieties. This is particularly evident for high-extractive containing residues such as culms, where the incomplete removal of extractives could be more problematic to obtain an acceptable summative mass closure. This may be confirmed by the fiber amounts obtained in leaf tissue, which showed similar amounts and rankings in the AIR (Table 4) and unextracted (Table 1) material as fewer extractives were lost compared to the culm tissues.

**Table 4:** Composition of untreated and pretreated alcohol-insoluble residue from sugarcane culm and leaf tissue

	FIJI_62	MQ239	Q124	Q208	QBN13-10020	SRA1	SRA5	FIJI_62	MQ239	Q124	Q208	QBN13-10020	SRA1	SRA5
	Culm							Leaf						
% Solid recovery														
HT	80.1 ± 1.8	79.9 ± 2.2	82.7 ± 5.5	84.1 ± 1.3	83.7 ± 1.1	76.2 ± 7.7	75.3 ± 1.5	78.6 ± 1.1	74.7 ± 4	74.5 ± 13	75.7 ± 4.9	79.1 ± 2	71.8 ± 4.3	76.3 ± 7.9
DA	83 ± 0.4	83.3 ± 0.3	84 ± 0.9	84.1 ± 0.3	85 ± 0.7	81.5 ± 0.2	82.3 ± 0.9	81.7 ± 2.4	79.8 ± 1.1	80.4 ± 2.3	81.1 ± 0.3	83.9 ± 0.4	80 ± 0.3	82.4 ± 0.7
IL	79 ± 2.2	84.3 ± 1.9	82.4 ± 2.1	76.8 ± 1.7	84.8 ± 0.5	89.7 ± 0.3	87.4 ± 2.2	77.5 ± 1.4	80.5 ± 1.5	77.1 ± 7.1	76 ± 0.3	78.5 ± 4.2	88 ± 0.4	79.4 ± 1.1
% Glucan														
Untreated	28.1 ± 0.6	22.5 ± 6	25.3 ± 2.8	26.3 ± 3.8	26 ± 2.6	29.3 ± 4.1	31.5 ± 7.7	19.8 ± 0.7	25.3 ± 5.3	26.9 ± 3.9	26.3 ± 2.3	20.6 ± 1.3	20.6 ± 1.9	22.2 ± 3.1
HT	34.3 ± 1.8	32.9 ± 3.2	35.5 ± 0.4	35.7 ± 2.2	35.7 ± 2.8	37.3 ± 1.1	36.1 ± 2.2	32.9 ± 0.7	30.4 ± 1.2	32.8 ± 0.7	34.5 ± 1	32.2 ± 2.9	36.4 ± 2.9	30.6 ± 4.7
DA	33.7 ± 2.2	32.2 ± 2.7	29.5 ± 1.3	36.9 ± 2.2	34.7 ± 2.6	44.6 ± 0.3	33.7 ± 2.7	33 ± 1.9	34.5 ± 1	34.3 ± 2.5	33.2 ± 5.5	34.2 ± 2.1	42.5 ± 1.9	29.9 ± 1.5
IL	36.1 ± 2	35.4 ± 2.1	39.5 ± 0.7	44.6 ± 2.6	37.9 ± 5.1	37.1 ± 2.7	42.4 ± 2.9	38.1 ± 0.8	38.4 ± 0.6	36.7 ± 0.2	39.4 ± 1	38.3 ± 4.7	39.2 ± 1.5	40.8 ± 0.9
% Xylan														
Untreated	20.3 ± 1.1	16.3 ± 3.1	17.7 ± 2.2	17.9 ± 2.5	18.1 ± 1.6	20.6 ± 2.6	20.8 ± 4	15.1 ± 0.6	19.5 ± 3.4	19.4 ± 2.3	18.1 ± 1.5	15.6 ± 0.5	14.9 ± 0.4	18.5 ± 2.2
HT	16.9 ± 1.1	16.1 ± 2.8	17.3 ± 0.7	16.3 ± 0.6	17.1 ± 1	13.1 ± 1.7	13.8 ± 3.1	16.9 ± 0.4	18.9 ± 0.8	18 ± 0.8	17 ± 0.3	16.3 ± 2.1	13.9 ± 0.6	15.9 ± 2.5
DA	16 ± 1.3	15.1 ± 0.5	12.2 ± 0.3	14.9 ± 0.3	14.4 ± 1.5	21.6 ± 0.2	13.8 ± 0.9	18.5 ± 1.3	19.5 ± 1.9	18.9 ± 1.6	17 ± 3.3	19.6 ± 1.3	22.5 ± 1.6	17.4 ± 1
IL	20 ± 0.8	20.4 ± 0.9	21 ± 0.3	21.2 ± 0.8	20.8 ± 1.5	20.6 ± 1.6	21.7 ± 0.8	21.9 ± 1	23.7 ± 0.2	21.8 ± 0.1	20.8 ± 0.5	22.2 ± 1.5	22 ± 1.2	23.6 ± 0.3
% AIL														
Untreated	18.3 ± 0.8	19.6 ± 0.1	18.3 ± 0.2	19.8 ± 0.4	18.5 ± 0.1	18.8 ± 0.8	19.2 ± 0.4	19.7 ± 5	14.9 ± 0.9	13.8 ± 1	15.2 ± 1.4	15.2 ± 0.6	14.7 ± 0.4	17.4 ± 1.3
HT	20.4 ± 1.5	20.4 ± 0.6	20.3 ± 2.3	20.5 ± 0.9	19.5 ± 0.3	22.7 ± 1.6	21.7 ± 1.7	19.9 ± 1.2	16.9 ± 1	14.9 ± 1.1	17.2 ± 0.4	16.9 ± 2.2	18.9 ± 0.4	20 ± 2.7
DA	20.6 ± 1.1	20.9 ± 0.3	20.5 ± 1.2	22.7 ± 1.9	20.3 ± 0.2	21.9 ± 0.2	21.3 ± 0.3	17.9 ± 0.7	17.1 ± 0.3	15.2 ± 0.1	16.5 ± 1.3	14.4 ± 0.2	15.8 ± 0.6	18.6 ± 0.6
IL	16.1 ± 0.7	19.4 ± 1.1	17.8 ± 1	14.6 ± 1.5	15.4 ± 1.1	19.6 ± 2.6	14.7 ± 0.7	12.6 ± 0.2	12.7 ± 0.1	14.5 ± 1.6	10.6 ± 0.6	10.5 ± 1.3	13.3 ± 1	12.9 ± 0.9
% ASL														
Untreated	2.7 ± 0.6	2.2 ± 0.2	3.1 ± 0.2	2.8 ± 0.4	3.2 ± 0.4	3.5 ± 0.3	3.4 ± 0.9	3.4 ± 0.3	3.8 ± 0.6	3.8 ± 0.5	3.7 ± 0.1	3.2 ± 0	3.2 ± 0.7	3.3 ± 0.2
HT	4.2 ± 0.1	4.1 ± 0.5	4.7 ± 0.1	4.5 ± 0.1	4.6 ± 0.2	4.7 ± 0.1	4.4 ± 0.4	5.1 ± 0.3	5.5 ± 0	5.3 ± 0.3	4.9 ± 0.2	5.2 ± 0.2	4.9 ± 0.4	5.4 ± 0.5
DA	3.9 ± 0.4	3.8 ± 0.1	3.8 ± 0.4	4.1 ± 0.1	4.1 ± 0.4	4 ± 0.1	3.8 ± 0.2	4.8 ± 0	5 ± 0.1	5.1 ± 0.1	4.6 ± 0.2	4.7 ± 0.3	4.8 ± 0.2	4.7 ± 0.2
IL	5 ± 0.2	4.9 ± 0.2	5.6 ± 0.2	6 ± 0.1	5.5 ± 0.4	5.5 ± 0.1	6.1 ± 0.3	6 ± 0.3	6.2 ± 0	6.2 ± 0	5.6 ± 0.1	6.1 ± 0.3	5.9 ± 0.3	5.5 ± 0.1
% Glucan conversion														
Untreated	32.7 ± 14.2	19.3 ± 4.4	22.3 ± 3.5	23 ± 6.5	15 ± 2	24.6 ± 5.8	18.1 ± 3.7	37.3 ± 1.5	36.9 ± 9.6	32 ± 3.9	31.9 ± 1.1	33.3 ± 1	48.2 ± 11	31.7 ± 5.1
HT	33.7 ± 10	24.2 ± 11.1	33.6 ± 10.2	26 ± 11.6	22.5 ± 8.1	50.5 ± 11.1	33.3 ± 16	47.1 ± 1.4	29.4 ± 3	44.6 ± 3.4	53.8 ± 5.2	60.8 ± 6.9	70.9 ± 5.5	22.8 ± 2.4
DA	26 ± 7.3	26.5 ± 13.4	37.4 ± 9.1	22.6 ± 1.6	16.2 ± 1.9	22.6 ± 2.2	21.1 ± 2.5	39.9 ± 1.1	39 ± 2.6	37.1 ± 1.1	40.8 ± 8.1	30.8 ± 1.5	32.3 ± 0.7	35.3 ± 3.4
IL	52.2 ± 17.8	19.1 ± 3.7	29.4 ± 6.8	50.6 ± 12.1	36 ± 3.2	41.4 ± 6.1	65 ± 5.2	72 ± 5.9	60.6 ± 9.1	42.3 ± 0.6	76.1 ± 17	60.3 ± 19.2	56.5 ± 8.4	76.4 ± 1.5

Means ± standard deviation

*HT* hydrothermal, *DA* dilute acid, *IL* ionic liquid, *AIL* acid-insoluble lignin, *ASL* acid-soluble lignin, *% Glucan conversion* glucan to glucose conversion after 26 h of pre-saccharification

### Effects of pretreatments on leaf and culm tissues

Solid recovery in the AIR samples following HT, DA and IL pretreatments was approximately 81% for both leaf and culm tissue indicating that about 19% of the biomass was solubilized (Table 4). About 32% xylan removal was detected in the culm tissues after HT and DA pretreatments (data not shown). In the leaf tissues, xylan removal averaged 27% and 8% in the HT and DA pretreatments, respectively. In contrast, the IL-pretreated material showed negligible change in xylan content. As expected, the greatest reduction in AIL was observed after the IL pretreatment which averaged 25.4% (culm) and 36.8% (leaf) AIL removal, while AIL removal in HT- and DA-pretreated samples averaged 12.0% and 6.8% for the culm and 13.7% and 16.7% for leaf tissues, respectively (data not shown). The highest AIL removal of 48.3% was obtained for the Fiji_62 leaf after IL pretreatment. These results are in agreement with the literature [12, 17, 25, 54–60] and indicate that the IL pretreatment predominantly results in lignin reduction, while xylan is the primary target in HT and DA pretreatments.

Biomass composition after pretreatment is shown in Table 4. There were small differences between genotypes in solid recovery and removal of lignin and xylan following pretreatment that resulted in changes in genotype and tissue rankings for the quantity of each fiber constituent after pretreatment. For example, xylan content in untreated samples was generally lower in leaf compared to culm tissues but after pretreatment, the opposite was found. Additionally, AIL content in Q124 leaf tissues was the lowest among the genotypes when samples were left untreated but was the highest after IL pretreatment. This finding is consistent with other reports [25, 27, 50, 61–63] and suggests that the tissues and genotypes varied in their susceptibility to each pretreatment likely resulting from their differing biomass compositions (Tables 1, 2, 3 and 4).

### Glucan conversion following pre-saccharification

**Fig. 1:**
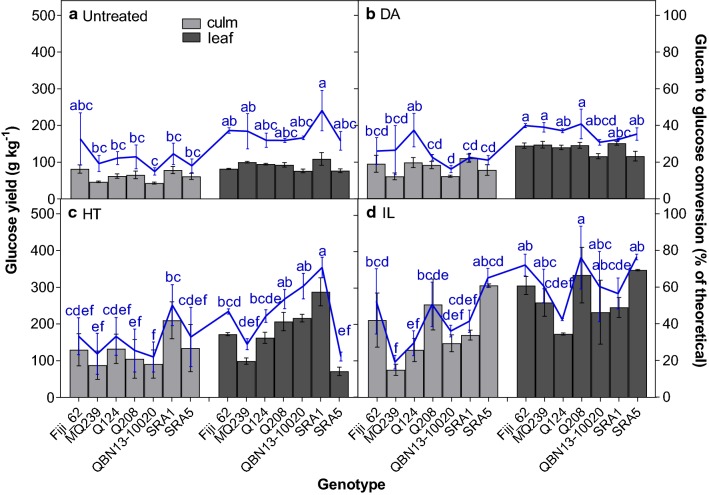
Glucose yield (bars) and percent conversion of glucan to glucose (line) of sugarcane culm (light bars) and leaf (dark bars) tissues after 26 h of pre-saccharification. Prior to saccharification, alcohol-insoluble residue samples were untreated (**a**) or subjected to dilute acid (DA) (**b**), hydrothermal (HT) (**c**) and ionic liquid (IL) (**d**) pretreatments. Least square adjusted means (untreated, *n* = 3; pretreated, *n* = 6) are presented ± standard deviation. Letters indicate differences in glucan conversion among genotype × tissue combinations within pretreatment according to Tukey’s honestly significant difference (*p *≤ 0.05)

Pretreated and untreated samples were pre-saccharified for 26 h and the ANOVA indicated that the effects of genotype, tissue, pretreatment and their interactions were significant for glucan conversion. Percent glucan conversion averaged over all genotypes after no treatment, DA, HT and IL pretreatments was 22.1%, 24.6%, 32% and 42% in culm tissues and 35.9%, 36.5%, 47.1% and 63.5% in leaf tissues for samples, respectively. These results demonstrate that the composition of pretreated samples and leaf tissues was more conducive to enzymatic saccharification than that in untreated samples and culm tissues, respectively (Fig. 1, Table 4). In agreement with the literature [25, 50], leaf tissues had glucan conversion that was approximately 1.5-fold of that in culm tissues. Since bagasse and leaf trash are generated at an equal rate [64] and have comparable cellulose content [25, 50], these results suggest that more than twice as much ethanol can be produced using both waste residues rather than solely bagasse.

Interestingly, the previous studies determined that straw had greater lignin content than bagasse implying that other factors were important in determining digestibility [25, 50]. Possibly due to variety or maturity differences between studies, these results were inconsistent with the current study. Untreated and pretreated leaves had approximately 13% less total lignin than the culm, in the present study. Their results are intriguing because they are in disagreement with the vast amount of literature proposing that lignin content plays the most central role in biomass degradability [30, 65–67]. These studies, however, have mostly focused on sugarcane bagasse and other biomass that is primarily composed of stem tissue, and therefore may have missed important relationships between biomass composition and saccharification efficiency that are present in leaf and other tissues.

Different saccharification efficiencies were observed for each pretreatment (Fig. 1, Table 4). The DA treatment did not significantly increase percent glucan conversion in either tissue (untreated culm, 22.1% and leaf, 35.9%; DA-treated culm, 24.6% and leaf, 36.5% averaged over genotype), despite the decrease in xylan and lignin that was comparable to that observed after HT pretreatment. The glucose yield averaged across genotypes increased, however, from 62.7 (culm) and 90.3 (leaf) g of glucose for each kg of biomass in untreated tissues to 86.3 g kg^−1^ and 138.6 g kg^−1^, due to the raised glucan content after DA pretreatment. The HT treatment had greater glucan conversion than the DA treatment (32% in the culm and 47% in the leaf), while the IL pretreatment produced the highest glucan conversion (42% in culm and 63.5% in leaf). The IL pretreatment may have been the most effective pretreatment because of its ability to perform well at relatively high solids loadings like that used in this study (20%) [17]. It also had the greatest reduction in AIL compared to the other pretreatments, removing about two- to threefold more AIL, which probably contributed to its success. Furthermore, 20% solids loading may have been too high for the conditions used during the DA treatment, thus resulting in the lack of improvement.

Genotypes responded differently depending on the pretreatment likely reflecting their varying levels of susceptibility to each pretreatment (Fig. 1, Table 4). For instance, Fiji_62, Q208 and SRA5 had the highest yields and glucan conversions for both tissue types following IL pretreatment, ranging from 50.6 to 76.4% conversion. SRA1 performed the best after the HT pretreatment possibly due to low xylan content. However, HT-pretreated SRA5 leaf tissue also had low xylan content, yet the percent glucan conversion was less than a third of that for SRA1 leaf tissues likely because it also had high AIL. For DA-pretreated samples, no differences in terms of sugar yield and glucan conversion were observed among genotypes for the leaf tissues, but when the culms were considered, percent glucan conversion was highest (37.4%) for Q124. For untreated samples, although no significant differences were observed between genotype within tissue, Fiji_62 and SRA1 appeared to have the highest glucan conversions for culm and leaf tissues, respectively. Moreover, biomass composition does not obviously explain why certain genotypes did well after a particular pretreatment. For example, the highest saccharification efficiency did not always correspond to genotypes with the lowest AIL or xylan. This suggests that a combination of many factors likely contribute to hydrolysis yield.

### Path analysis for the biomass components that affect glucan conversion after pre-saccharification

**Fig. 2:**
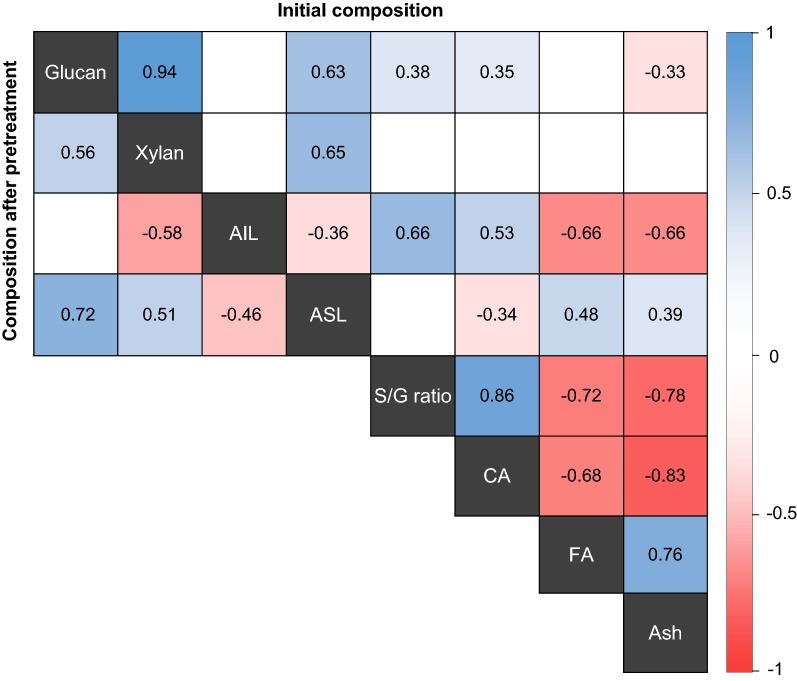
Pearson correlation coefficients for biomass traits in sugarcane leaf and culm tissues measured on alcohol-insoluble residue (AIR) using initial and pretreated composition values. Cells with text have significant correlations (*p *≤ 0.05) and blank cells are non-significant. S/G ratio, CA, FA and ash were not measured after pretreatment. AIL: acid-insoluble lignin; ASL: acid-soluble lignin; S/G: syringyl to guaiacyl ratio; CA: *p*-coumaric acid; FA: ferulic acid

Path analysis was utilized to elucidate the relationships between glucan conversion after pre-saccharification and biomass composition (Table 5). This analysis was used because there were numerous strong correlations observed among the biomass traits (Fig. 2) which can confound the true relationships between these traits and glucan conversion and path analysis is able to determine individual contributions of traits in a complex interacting systems such as this. Both initial and pretreated composition values were used in separate path analyses since the change in composition after pretreatment varied between samples (Table 4).

**Table 5:** Path analysis direct effects of biomass traits on glucan conversion after 26-h pre-saccharification

Trait	Direct effect	
	Before pretreatment^b^	After pretreatment
Glucan^a^		0.25
Xylan	− 0.44	− 0.50
AIL	− 0.10	− 0.65
ASL	0.43	0.30
S/G ratio	− 0.38	
CA	− 0.15	
FA	− 0.11	
Ash	− 0.01	
Residual effect	0.64	0.71
*R* ^2^	0.59	0.49

AIL: acid-insoluble lignin; ASL: acid-soluble lignin; S/G ratio: syringyl to guaiacyl ratio; CA: *p*-coumaric acid, FA: ferulic acid

^a^Glucan was removed from the “Before pretreatment” analysis because it was a major source of multicollinearity

^b^“Before pretreatment” analysis was based on initial composition values and “After pretreatment” analysis was based on pretreated composition values (including the untreated treatment group)

Severe multicollinearity between variables in path analyses often leads to unreliable path coefficients [68]; so, several tests were performed prior to the analysis to determine if it was present. Multicollinearity was detected in the analysis using initial composition values. This was determined because variance inflation factors were > 10 for two variables, the condition number (ratio between smallest and largest eigenvalues of the correlation matrix) was 140 and the correlation matrix determinant (product of eigenvalues) was 1.57 × 10^−4^ [68]. In the analysis of eigenvalues and eigenvectors, glucan was identified as having the largest weight (0.76) associated with the smallest eigenvalue (0.03) indicating that it was a major source of multicollinearity and therefore, it was excluded from the model that was based on initial composition values. After pretreatment, multicollinearity was not present between variables; so, all variables were included in the model that was based on pretreated values.

Using the initial composition values, xylan (− 0.44) had the strongest negative effect on glucan conversion, followed by S/G ratio (− 0.38), CA (− 0.15), FA (− 0.11) and AIL (− 0.10) indicating that breeding to reduce these factors may improve conversion efficiency (Table 5). ASL had a positive effect (0.43); whereas the effect of ash content was negligible. Similarly when pretreated values were considered, negative effects were observed for xylan (− 0.50) and AIL (− 0.65) and a positive effect was seen with ASL (0.30) and glucan (0.25).

The negative effects of xylan and AIL on glucan conversion are consistent with the recalcitrance model often described in the literature, whereby hemicellulose and lignin form a physical barrier obstructing the hydrolytic enzymes from accessing the cellulose. Furthermore, hydroxycinnamic acids, FA and CA, are also known to play a role in recalcitrance [69]. They are responsible for cross-linking xylan and lignin further strengthening the cell wall matrix [42, 70], which is consistent with the negative association with glucan conversion observed for initial values in this study. However, their effects on glucan conversion were minimal compared to the other factors (Table 5), suggesting that modifying these traits may not achieve much improvement in conversion rates. Interestingly, another study found that etherified FA and CA were negatively correlated with saccharification yields in sugarcane, while esterified FA and CA were positively correlated [23]. Thus, the opposite effects of the two types of covalent bonds linking FA and CA to the lignin–polysaccharide matrix may cancel each other out when they are quantified as totals, as in the current study, and this may have been the reason the effects were small.

The positive effect of glucan content (0.25) of pretreated biomass on percent glucan conversion is not surprising and it likely reflects a greater likelihood of cellulose enzymes hydrolyzing glucan when there is more available (Table 5). ASL also had a strong positive association with glucan conversion using both initial (0.43) and pretreated (0.30) values providing evidence that this may be an important target for breeding. ASL is the fraction of lignin that is soluble in concentrated sulfuric acid during the Klason lignin determination procedure. It is made up of low-molecular-weight lignin degradation products and water-soluble lignin–carbohydrate compounds formed with hemicellulose monosaccharides during Klason analysis [71–73]. Previous studies have demonstrated that the most predominant linkage found in lignin, the aryl-ether β-*O*-4 bond, is cleaved under acidic conditions [74] suggesting that the types of biomass that yielded a high ASL content have lignin that is rich in β-*O*-4 bonds. Pretreatments such as DA and HT create acidic environments in the biomass slurry and thus catalyze β-*O*-4 cleavage [75, 76]. Therefore, it is not surprising that biomass which has a high proportion of these bonds would be more susceptible to pretreatment and in turn have higher hydrolysis yields. In this regard, quantification of ASL may have potential use for screening genotypes for lignin composition that is vulnerable to pretreatment and results in increased glucan conversion.

Additionally, previous research has shown that ASL is primarily composed of syringyl lignin [71, 73, 77] because the S units are more easily fragmented than G units during the Klason lignin procedure. This is most likely due to the high proportion of β-*O*-4 linkages present [78]. In accordance, Nawawi et al. [78] found that ASL and syringyl content were positively correlated. The results of these previous studies and the observation of a positive association between conversion efficiency and ASL in the present study suggest that high S/G ratio would be advantageous for improving glucan conversion. On the contrary, no correlation was observed between ASL and S/G content (Fig. 2) in the present study, and the latter had a negative direct effect (− 0.38) on glucan conversion suggesting that a low S/G ratio is favorable (Table 5). The negative association likely resulted because low S/G content and high glucan conversion coincided in the leaves; whereas culm tissues had relatively high S/G ratios and low glucan conversion. Additionally, a recent study has shown that S units are more highly cross-linked to xylan than G units [10] which could explain why a lower S/G ratio resulted in reduced recalcitrance. In agreement with these results, Davison et al. [79] and Papa et al. [63] also observed that low S/G led to high hydrolysis rates in *Populus* spp. and *Eucalyptus globulus*.

There were limitations in this study despite the strong relationships observed between biomass composition and saccharification efficiency. For instance, S/G ratio, CA, FA and ash were not measured after pretreatment, which hinders the conclusions reached for the effect of pretreated composition on conversion efficiency. Furthermore, coefficient of determinations (*R*^2^) for both analyses was fairly low and indicated that 59% and 49% of the variance in glucan conversion are explained by the variables and dataset for initial and pretreated compositions, respectively. This indicates that there were other factors that were not measured in this study which have a substantial impact on conversion efficiency and should be explored in future research.

### Ethanol yield and glucan to ethanol conversion after SSF

**Fig. 3:**
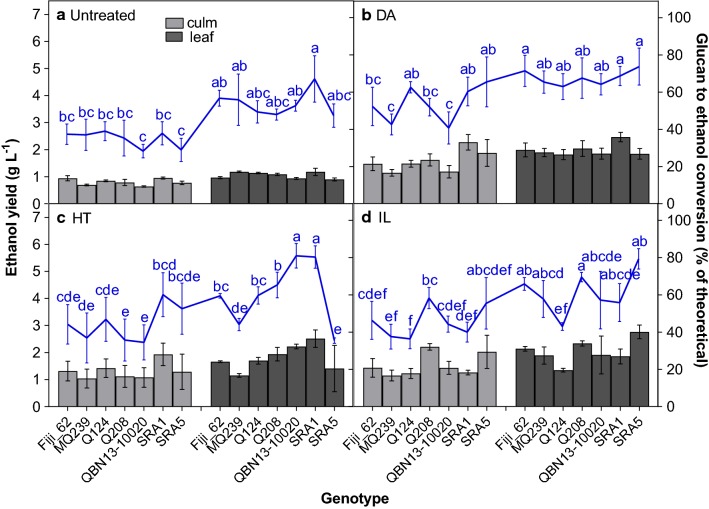
Ethanol yield (bars) and percent conversion of glucan to ethanol (line) in sugarcane culm (light bars) and leaf (dark bars) tissues after 15 h of SSF. Prior to SSF, alcohol-insoluble residue samples were untreated (**a**) or subjected to dilute acid (DA) (**b**), hydrothermal (HT) (**c**) and ionic liquid (IL) (**d**) pretreatments and then pre-saccharified for 26 h. Least square adjusted means (untreated, *n* = 3; pretreated, *n* = 6) are presented ± standard deviation. Letters indicate differences in glucan conversion among genotype × tissue combinations within pretreatment according to Tukey’s honestly significant difference (*p *≤ 0.05)

**Fig. 4:**
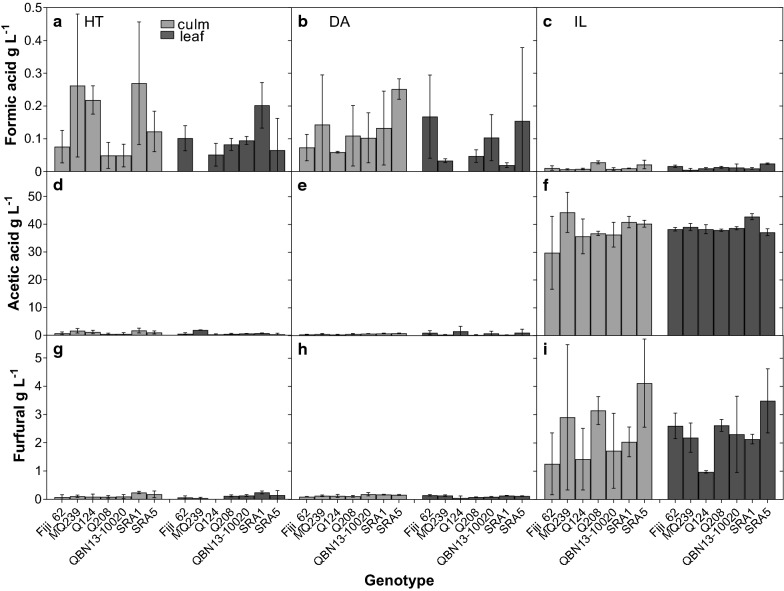
Fermentation inhibitory compounds present in sugarcane culm (light bars) and leaf (dark bars) alcohol-insoluble residue samples after hydrothermal (**a**, **d**, **g**), dilute acid (**b**, **e**, **h**) and ionic liquid (**c**, **f**, **i**) pretreatments. Means (*n* = 3) are presented ± standard deviation

Genotype, tissue, pretreatment and their interactions were significant for the percent glucan to ethanol conversion after 15 h of SSF. Averaged over all genotypes, glucan to ethanol conversion was 35.7%, 53.7%, 44.9% and 45.5% in culm tissues and 54.7%, 67.7%, 61.3% and 61.2% in leaf tissues after no pretreatment and DA, HT and IL pretreatments, respectively. Interestingly, the rankings for overall pretreatment performance were not the same after SSF as they were after pre-saccharification. For instance, the IL pretreatment was no longer the highest ranking but rather, the DA pretreatment which was the least successful after pre-saccharification (Fig. 1) had the greatest percent glucan conversion after SSF (Fig. 3). One explanation for the unexpected low ethanol yields obtained in IL-pretreated material is the presence of fermentation inhibitory compounds formed during pretreatment from the breakdown of the material. Acetic acid and furfural were detected at concentrations of ~ 40 g L^−1^ and 2.4 g L^−1^ in IL-treated samples while DA- and HT-treated samples had < 1 g L^−1^ and < 0.15 g L^−1^, respectively (Fig. 4). About 80% of the acetic acid present in these samples came directly from the IL, ethanolamine acetate, itself, since the procedure was done on a one-pot basis [17], where the IL was not washed from the sample, but diluted to about 7 wt% after pretreatment. The remainder of the acetic acid was probably derived from the deacetylation of the hemicellulose during pretreatment and the furfural likely arose from the degradation of xylose [80]. Therefore, although the IL pretreatment showed promise during pre-saccharification, the pretreatment conditions should be optimized for sugarcane to decrease the presence of inhibitory compounds.

Analogous to the results for glucan conversion after pre-saccharification (Fig. 1), pretreated and leaf tissues had higher percent conversion of glucan to ethanol compared to untreated and culm tissues, respectively (Fig. 3). Additionally, the rankings for genotype performance in conversion of glucan to ethanol were almost identical to those observed after pre-saccharification (Fig. 1). Furthermore, glucan conversion after pre-saccharification and SSF were significantly positively correlated (*r *= 0.69). These results are consistent with the literature [62, 66, 81, 82] and indicate that ethanol yields are greatly dependent on the efficiency of saccharification. Therefore, improving saccharification efficiency by modifying the key characteristics identified in the path analysis should result in increased ethanol production from sugarcane biomass.

## Conclusion

Identifying biomass characteristics that increase susceptibility to saccharification is an important goal to breed bioenergy varieties of sugarcane. In this study, leaf and culm tissues from seven sugarcane genotypes with varying biomass compositions were assessed for their efficiency of converting glucan to glucose. Additionally, several pretreatments were applied to understand the relationship between glucan conversion and composition that arises after pretreatment. Overall, there was no genotype that was superior to all others across all pretreatments suggesting that biomass resistance to saccharification changes depending on the pretreatment. Path analysis determined that AIL and xylan content had the strongest negative influences on glucan conversion supporting the recalcitrance model often reported in the literature. Interestingly, S/G ratio also had a negative effect and ASL content had a positive effect on glucan conversion indicating that modifying lignin composition will be key for developing improved biomass varieties. Furthermore, trends observed across genotypes and tissues in glucan conversion after pre-saccharification corresponded to those after SSF indicating that improving saccharification efficiency will result in greater ethanol yields.

## Methods

### Plant material and biomass preparation

Seven *Saccharum* spp. genotypes (Fiji_62, MQ239, Q124, Q208, QBN13-10020, SRA1 and SRA5) varying in fiber and sugar content were selected for this experiment. All the genotypes are complex *S. officinarum* × *S. spontaneum* commercial hybrids except for FIJI_62 (*S. officinarum*) and QNB13-10020 (commercial hybrid × *S. spontaneum*). FIJI_62 and QNB13-10020 were selected because they were expected to represent extreme and contrasting phenotypes characteristic of the progenitors (e.g., high sugar, low fiber vs low sugar, high fiber) from which the commercial hybrids are derived. Genotypes were grown in the Sugar Research Australia field trials at the Meringa Station and 12-month-old plants were harvested in November 2017 in triplicate with each replicate consisting of two stalks from the same plant. Leaves were removed and oven-dried. Green tops were discarded and the remaining stalk was shredded and dried. Samples were then ground with a knife mill (Polymix ^®^ PX-MFC 90 D; Kinematica, Lucerne, Switzerland) through a 0.5-mm screen.

### Extractives and starch removal

Sugars and other extractives were removed by solvent extraction using the following steps. Six gram sub-samples were extracted in 30 mL of 100% ethanol at 70 °C for 20 min. Samples were then washed with 30 mL of a 2:3 v/v methanol and chloroform solution, respectively, with shaking overnight. Samples were again washed with 30 mL of a 2:3 v/v methanol chloroform solution, shaken for 45 min. Sequential extractions using 30 mL of 100% ethanol, 65% ethanol, 2 × 80% ethanol, and 2 × 100% ethanol followed. At each step, 1-mL aliquots of supernatant were retained and sucrose, fructose and glucose were determined by HPLC. The extractives were quantified by evaporating 1 mL of supernatant with nitrogen gas and weighing the remaining solid. The AIR was oven-dried at 50 °C for 36 h and then destarched to ensure that starch present in the samples would not be included in the glucan quantification during the compositional analysis [83]. Destarching was achieved using the following method, as described by Harholt et al. [84] with adaptation. For each sample, approximately 3 g of AIR was suspended in 30 mL of a 0.1-M potassium phosphate buffer solution, pH 6.3, containing 1-mM calcium chloride, that had been preheated to 95 °C. Then, 90 U of α-amylase (Megazyme, Wicklow, Ireland) was added and the samples were incubated at 85 °C for 30 min. After the samples cooled to room temperature, 90 U of amyloglucosidase (Megazyme) and 45 U of pullulanase (Megazyme) were added and the sample was shaken overnight at 25 °C. After centrifugation at 4000 rpm for 10 min, the supernatant was removed and the pellet was washed with 30 mL of the 1-mM calcium chloride, 0.1-M potassium phosphate buffer solution. Subsequent washes with 30 mL of 96% ethanol, and 2 × 70% ethanol followed. The destarched AIR samples were freeze-dried and stored at room temperature for further analysis.

### Compositional analysis

The ash content of the raw biomass was determined using a muffle furnace (Isotemp 650-14, Fisher Scientific) heated to 575 °C with a temperature ramp [85]. Fiber composition of the starch-free AIR and pretreated solids was determined according to the two-step sulfuric acid hydrolysis procedure from National Renewable Energy Laboratory (NREL) (NREL/TP-510-42618) [86]. One hundred or 300 mg of biomass mixed with 1 or 3 mL of 72% sulfuric acid, respectively, was incubated at 30 °C for 1 h, then diluted to 4% sulfuric acid and autoclaved for 1 h at 121 °C. ASL in the liquid fraction of the hydrolysate was quantified by measuring the UV absorption at 240 nm using a UV–Vis spectrophotometer (Nanodrop 2000; Thermo Fisher Scientific). Glucose and xylose concentration, in the liquid fraction, was determined by HPLC and anhydro correction factors of 0.9 and 0.88 were used to calculate glucan and xylan content, respectively. After vacuum filtration, AIL was determined as the sample weight after heating the solid fraction at 105 °C overnight less the ash resulting after incineration at 575 °C in a muffle furnace (Isotemp 650-14, Fisher Scientific). The percentage of glucan, xylan, AIL and ASL determined in each of the AIR samples was used to calculate the amounts present in the original, dry material prior to extraction according to Eq. 1:

1 $${\text{\% unextracted amount}} = \left( {{\text{\% amount in AIR}}/100} \right) \times \left( {100 - {\text{\% extractives}}} \right)$$

MLG was quantified in raw biomass samples using the Megazyme MLG assay kit and procedures.

The release of CA and FA from plant cell walls was quantified by treatment with sodium hydroxide solution described by Santiago et al. [87] modified. Briefly, 10 mg of the cell wall-bound material was weighted using the Joint BioEnergy Institute Biomass Preparation System Robot created by Labman Automation Ltd. (North Yorkshire, UK), at a target mass of 10 mg of biomass into a 1.4-mL 96-well Micronic Rack. The samples were covered with Micronic TPE Push Caps and incubated overnight in the dark with shaking at 130 rpm at 30 °C, with 0.5 mL of 2-M sodium hydroxide. Then, 0.1-mL 6 N hydrochloric acid was added to lower the pH to 2.0. Supernatants were extracted three times with ethyl acetate (0.5 mL each). The collected organic fractions were combined and reduced to dryness using a nitrogen flow. The final extract was dissolved in 0.3 mL of HPLC-grade acetonitrile, filtered through a 96-well 0.45-μm filter plate (Whatman 7700-1301 http://www.gelifesciences.com) by centrifugation (3000×*g* for 3 min), and stored at − 20 °C until HPLC analysis.

### Determination of the S/G ratio by py–GC/MS

The S/G ratio was determined in the starch-free AIR by pyrolysis coupled with gas chromatography–mass spectrometry (py–GC/MS), as described by Ralph and Hatfield [88]. Sub-samples of 0.5 mg were pyrolyzed at 550 °C using the pyroprobe 5200 (CDS Analytical, Inc., Oxford, PA, USA) connected to a gas chromatography mass spectrometry (GC/MS) system (Agilent 6890) composed of a Trace GC Ultra and a Polaris-Q MS (Thermo Electron Corporation, Waltham, MA, USA) equipped with a TR-SMS column (60 m 0.25 mm ID 0.25 lm) and operated in split mode (40 mL min^−1^) using He as carrier. The chromatograph program was set as follows: 5 min at 50 °C, followed by an increase of 5 °C min^−1^ to 300 °C, finally maintained at 300 °C for 5 min. Pyrolysis products were identified on the basis of their mass spectra using the NIST08 mass spectrum library (Table 3). Compounds of S, G and H origin were quantified from the pyrogram using the peak area. The S/G ratio was calculated as the sum of all peak areas of S molecules divided by the sum of all peak areas of G molecules; 4-vinylguaiacol and 4-vinylphenol were detected but, since these compounds are largely released from ferulates and *p*-coumarate esters in grass species [53, 89], they were omitted from the lignin monomer estimation.

### Determination of cellulose crystallinity

Cellulose crystallinity was characterized with powder X-ray diffraction on one biological replicate from each genotype for the culm and from genotypes representing extremes for fiber content (Fiji_62, QBN13-10020 and SRA1) for the leaf. The data were collected according to the method of Cruz et al. [18] with a PANalytical Empyrean X-ray diffractometer equipped with a PIXcel^3D^ detector and operated at 40 kV and 40 kA using Cu *Kα* radiation (*λ* = 1.5418 Å). The patterns were collected in the 2*θ* range of 5–65° with a step size of 0.026°, and an exposure time of 300 s. A reflection–transmission spinner was used as a sample holder and the spinning rate was set at 4 rpm throughout the experiment. The crystallinity index (CrI) was determined from the crystalline (*A*_cr_) and amorphous peak (*A*_am_) areas of the measured diffraction patterns using the software package HighScore Plus^®^ according to Eq. (2):

2 $${\text{CrI}} = \frac{{\sum A_{{{\text{cr}} .}} }}{{\sum A_{{{\text{cr}} .}} + \sum A_{{{\text{am}} .}} }}$$

### Pretreatments

Three pretreatments (HT, DA and IL) were applied to the biomass prior to saccharification. All pretreatments were performed at 20% solids loading using 0.6 g of starch-free AIR mixed with 2.4 g of solution in pressure tubes. This solids loading was selected because it was considered suitable across the range of pretreatments and economically attractive for an industrial biorefinery [17, 80]. Pretreatment conditions are shown in Table 6. The optimal temperature and duration, determined from literature reports [12–14, 17, 90, 91], was applied for each pretreatment. Following HT and DA pretreatments, biomass was removed from the tubes by washing with 15 mL of DI water. The pretreatment using biocompatible protic IL ethanolamine acetate, without pH adjustments, water-wash and solid–liquid separations was performed on a “one-pot” basis [17]. Doing so, 250 mg (equivalent to 50-mg dry biomass) of the IL-pretreated slurry was removed and maintained in 15-mL 24-deep-well polypropylene plates at 4 °C for 5 h until saccharification. The remaining slurry was washed from the pressure tube with 15 mL of water and then washed thrice with 40 mL of water to remove the IL for further analysis. Samples were centrifuged at 4000 rpm for 10 min and 1 mL of supernatant was collected for quantification of fermentation inhibitory products by HPLC according to NREL protocol (NREL/TP-510-42623) [92]. After removing the supernatant, the samples were freeze-dried and the percent solid recovery was determined gravimetrically (i.e., calculated as the dry mass recovered as pretreated solids). Additionally, compositional analyses were performed on lyophilized, pretreated samples, as described previously.

**Table 6:** Pretreatment conditions

Pretreatment	Solvent	Apparatus	Temperature (°C)	Duration (min)
HT	Water	Sand bath	180	20
DA	0.1 M sulfuric acid	Autoclave	120	60
IL	Ethanolamine acetate	Oil bath	160	30

*HT* hydrothermal, *DA* dilute acid, *IL* ionic liquid

### Simultaneous saccharification and fermentation

Pre-saccharification was performed on starch-free AIR samples that were pretreated or left untreated, in 15-mL 24-deep-well polypropylene block sealed with a peelable heat seal, applied using a PlateLoc sealer (175 °C, 4 s) (Agilent Technologies) according to NREL protocol (NREL/TP-5100-63351) [93] and Sun et al. [17] with modifications. For consistency with the IL treatment, 50-mg samples from HT- and DA-pretreated biomass (dry basis) were mixed with 200 µL of water prior to pre-saccharification. The slurry was incubated at 50 °C for 26 h in 3.08 mL of 50-mM citrate buffer (pH 5) containing an enzyme mixture of 9:1 v/v Ctec3 and Htec3 enzymes (Novozymes, Franklington, NC, USA), respectively, at a concentration of 10 mg g^−1^ biomass with constant agitation at 800 rpm. At 26 h, 80 µL of supernatant was removed from the reactions for determination of glucose yield. Percentage glucan conversion was calculated, as previously described by Healey et al. [94], as the amount of glucose produced after 26-h saccharification divided by the theoretical amount of glucose produced based on the original glucan present in the AIR sample and then multiplied by 100.

Simultaneously, *Saccharomyces cerevisiae* strain BY4741 (MATα his3Δ0 leu2Δ0 met15Δ0 ura3Δ0) was grown in 2% YPD media at 30 °C and shaken at 200 rpm. After 24 h, the culture broth had an optical density at 600 nm of 4 and the yeast cells were collected by centrifugation at 3220 rpm for 5 min and washed thrice with 0.2% sterile peptone solution and suspended in 0.1-M phosphate buffer solution. The yeast was added to the samples after 26 h of pre-saccharification, plates were resealed, and simultaneous saccharification and fermentation were conducted at 37 °C for 15 h with constant agitation at 250 rpm. Supernatant aliquots of 80 µL were collected 15 h after addition of the yeast. Ethanol production was determined by HPLC. Percent glucan to ethanol conversion was calculated as the amount of ethanol produced after 15-h SSF divided by the theoretical amount of ethanol produced based on the original glucan present in the AIR sample and then multiplied by 100. All assays on the three biological replicate pretreated materials were performed in duplicate trials.

### Determination of glucose after pre-saccharification

Quantification of glucose after 26 h of pre-saccharification was performed using YSI 2700 Biochemistry Analyzer (Xylem, Inc., Yellow Springs, OH, USA). An automatic calibration was performed using glucose/lactate calibrator YSI 2776 (2.50 g L^−1^ glucose) and the linearity was tested prior to run with YSI 1531 (9.00 g L^−1^ glucose) linearity standard. The YSI glucose oxidase (Glucose Oxidase Membrane Kit, YSI 2365) and xylose oxidase (Xylose Oxidase Membrane Kit, YSI 2761) was used and the YSI 2700 analyzer measured the glucose with aspiration of 35 µL of sample. The samples were automatically flushed from the electrode chamber within 30 s using YSI 2357 Buffer Concentrate Kit.

### High-performance liquid chromatography (HPLC) for determination of sugars, coumaric acid, ferulic acid, ethanol and inhibitory products

Ethanol was quantified using a HPLC Agilent 1260 Infinity system (Agilent, Santa Clara, CA, USA) with a Bio-Rad 300 × 7.8 mm Aminex 87 H column (Bio-Rad, Hercules, CA, USA) with a BioRad cation H guard column. The Agilent 1260 refractive index detector was held at 35 °C. The samples were run using an isocratic 4-mM sulfuric acid eluent at 0.6 mL min^−1^ and 60 °C for 16 or 45 min. Sucrose (non-reducing sugar), glucose, and fructose (reducing sugars) were quantified using a 10 mM sulfuric acid eluent at a flow rate of 0.3 mL min^−1^ at 18 °C for 22 min.

HPLC separation of lignin-derived aromatics (coumaric acid and ferulic acid) was performed on an Agilent Eclipse Plus Phenyl-Hexyl, (5 µm, 250 mm, 4.6 mm). The mobile phase consisted of 0.07% formic acid and 10-mM ammonium acetate in aqueous phase (A) and 10-mM ammonium acetate in 90% acetonitrile (B). The elution program was as follows: 0 min, 0.5 mL min^−1^, 30% B; 12 min, 0.5 mL min^−1^, 80% B; 12.1 min, 0.5 mL min^−1^, 100% B; 12.8 min, 1 mL min^−1^ 30% B, then continued to 15.6 min at 1 mL min^−1^ and the column was held at 50 °C.

Sugar calibration standards were prepared and diluted to create six-point calibration curve, 0.0156–2.0 mg mL^−1^ for xylose and 0.03125–4.0 mg mL^−1^ for glucose, 0.325–20 mg mL^−1^ for fructose, glucose, and sucrose. CA and FA were prepared and diluted in acetonitrile to create six-point calibration curve, 1 g L^−1^–0.12 mg mL^−1^. Standards were run at the beginning and end of each 96 well plate. De-ionized water blanks were inserted into the sample queue before and after each run of standards. The concentration of analytes of interest from samples was calculated using the Chemstation software package and integrating the area under each compound detection peak.

### Statistical analyses

All statistical analyses were conducted using Statistical Analysis Software (SAS, version 9.4; SAS Institute, Cary, NC). All data were subjected to a Lund’s test of studentized residuals to detect outliers, which were removed prior to mean calculations and further analyses. Analysis of variance (ANOVA) was used to compare glucan conversion means across genotypes and tissues. Data were fit to a generalized linear mixed model using PROC GLIMMIX with genotype, tissue and their interaction as fixed effects and repetition as a random effect. The assumptions of normality, homogeneity, and random distribution of error were tested using the Shapiro–Wilk statistic, Levene’s test, and by visual analysis of residual plots, respectively. Based on the results of these tests, a covariance structure, which had heterogeneous error, was specified as necessary. Pearson correlation coefficients were determined among biomass traits and glucan conversion using PROC CORR. Scatterplots were observed to ensure variables had linear relationships and bivariate normal distributions.

Path analysis was conducted to elucidate the associations of the biomass traits with glucan conversion. Prior to path analysis, the presence of multi-collinearity between variables was assessed by computing the condition number, correlation matrix determinant, variance inflation factors, and eigenvalues as well as associated eigenvectors as described by Olivoto et al. [68]. Path analysis was then conducted using initial and pretreated biomass composition values with PROC IML as described by Kang [95]. The dependent variable for the analysis using initial composition was glucan conversion averaged over all pretreatments for each genotype × biological replicate × tissue combination. The dependent variable for the analysis using pretreated composition was glucan conversion averaged over technical replicates for each genotype × biological replicate × tissue × pretreatment combination. Independent variables included were all measured variables except those identified as collinear, MLG because it was likely washed away during sample extraction and thus not present during pre-saccharification and cellulose crystallinity because measurements were made on a subset of samples.

## Data Availability

The datasets used and analyzed in the current study are available from the corresponding author on reasonable request.

## Abbreviations

*A*_am_: amorphous peak area
*A*_cr_: crystalline peak area
AIL: acid-insoluble lignin
AIR: alcohol-insoluble residue
ANOVA: analysis of variance
ASL: acid-soluble lignin
CA: *p*-coumaric acid
CrI: crystallinity index
DA: dilute acid
FA: ferulic acid
GC/MS: gas chromatography mass spectrometry
H: *p*-hydroxyphenyl
HPLC: high-performance liquid chromatography
HT: hydrothermal
IL: ionic liquid
MLG: mixed linkage β-d-glucan
NREL: National Renewable Energy Laboratory
SAS: Statistical Analysis Software
S/G: syringyl/guaiacyl
SSF: simultaneous saccharification and fermentation
